# Model-driven elucidation of the inherent capacity of *Geobacter sulfurreducens* for electricity generation

**DOI:** 10.1186/1754-1611-7-14

**Published:** 2013-05-29

**Authors:** Longfei Mao, Wynand S Verwoerd

**Affiliations:** 1 Centre for Advanced Computational Solutions, Wine, Food & Molecular Bioscience Department, Lincoln University, Ellesmere Junction Road, Lincoln, 7647, New Zealand

**Keywords:** MFC, Microbial fuel cell, Geobacter sulfurreducens, Bioelectricity, Flux balance analysis, Flux variability analysis, Flux minimization, FATMIN

## Abstract

**Background:**

*G*. *sulfurreducens* is one of the commonest microbes used in microbial fuel cells (MFCs) for organic-to-electricity biotransformation. In MFCs based on this microorganism, electrons can be conveyed to the anode via three ways: 1) direct electron transfer (DET) mode, in which electrons of reduced c-type cytochromes in the microbial outer membrane are directly oxidized by the anode; 2) mediated electron transfer (MET) mode, in which the reducing potential available from cell metabolism in the form of NADH is targeted as an electron source for electricity generation with the aid of exogenous mediators; and 3) a putative mixed operation mode involving both electron transfer mechanisms described above (DET and MET). However, the potential of *G*. *sulfurreducens* for current output in these three operation modes and the metabolic mechanisms underlying the extraction of the reducing equivalents are still unknown.

**Results:**

In this study, we performed flux balance analysis (FBA) of the genome-scale metabolic network to compute the fundamental metabolic potential of *G*. *sulfurreducens* for current output that is compatible with reaction stoichiometry, given a realistic nutrient uptake rate. We also developed a method, **f**lux variability **a**nalysis with **t**arget flux **mi**nimization (FATMIN) to eliminate futile NADH cycles. Our study elucidates the possible metabolic strategies to sustain the NADH for current production under the MET and Mixed modes. The results showed that *G*. *sulfurreducens* had a potential to output current at up to 3.710 A/gDW for DET mode, 2.711 A/gDW for MET mode and 3.272 A/gDW for a putative mixed MET and DET mode. Compared with DET, which relies on only one contributing reaction, MET and Mixed mode were more resilient with ten and four reactions respectively for high current production.

**Conclusions:**

The DET mode can achieve a higher maximum limit of the current output than the MET mode, but the MET has an advantage of higher power output and more flexible metabolic choices to sustain the electric current. The MET and DET modes compete with each other for the metabolic resource for the electricity generation.

## Background

*Geobacter sulfurreducens* has been extensively studied for microbial fuel cell (MFC) applications since discovered in 1994 [1]. The electrons in the metabolism of *G*. *sulfurreducens* can be converted to a comparatively large amount of electrical energy through the direct electron transfer (DET) established between reducing processes and the MFC electrode. DET relies on membrane bound c-type cytochromes and electrically conducting nanowires [2], and allows MFCs based on this microbe to obviate the need to replenish exogenous mediators. DET has been suggested to resemble mechanisms observed for electron transport to Fe(III) citrate [3].

Although *G*. *sulfurreducens* do not excrete redox shuttles [4,5], exogenous redox shuttles can be added to pull electrons from the reduced intracellular carriers (e.g., NADH) to sustain power generation in MFCs in a mediated way [5]. The electron shuttle compounds allow bacteria to utilise a remote electron acceptor (anode) that is not directly accessible to the cells, enhancing the electron transfer efficiency between the cellular metabolism and the electrode [6]. Therefore, the mediated electron transfer (MET) mode could also be advantageous for some MFCs in which the microbes are not grown on the anode.

For the mediated electron generation process, the intracellular electron-shuttling compound NADH has received considerable attention, since it is used to accept the electrons released from an organic substrate and donates them to various oxidoreductase enzymes and electron acceptors (such as quinone and ferricyanide) in the respiratory electron transfer chain of all living cells [7-12]. Exogenous mediators, such as bromocresol green (BG) and neutral red (NR), may target any steps of NADH regeneration in the intracellular electron-transfer pathways in cells and convey the electrons to an extracellular electrode (anode) [13-15]. Besides, it has been proposed that the theoretical limit of MFC voltage output is the potential difference between NADH and the reaction in the cathode if microorganisms are used as a biological catalyst [10,16]. Therefore, targeting NADH as the electron source in MFCs of MET mode can liberate the maximum power achievable for a microorganism.

Furthermore, a putative mixed operation mode (Mixed mode) involving both the DET and MET may exist when the cells are grown on the anode and the redox shuttles are added to the anodic reactor of the MFC. The putative Mixed mode may not be practical since different anode potentials could be required to accept the electrons from c-type cytochrome and the reduced mediators, but study of the Mixed mode can help elucidate the metabolic connection between the MET and DET modes.

Scanning the previous literature regarding MFCs based on *G*. *sulfurreducens* indicates that there are two questions needed to be answered: 1) What is the maximum potential of *G*. *sulfurreducens* for current output in MFCs under the three aforementioned operation modes? 2) Since a variety of reactions inside a cell are associated with NADH regeneration, what is the best metabolic strategy to choose NADH supply reactions, to maximize the current output in the MET and Mixed modes?

To address these two issues, in this study, we employed flux balance analysis (FBA) to examine the maximum amperage output of metabolic-driven electricity generation based on *G*. *sulfurreducens* in three electricity generation modes, namely, the c-type cytochrome dependent DET mode, the NADH-dependent MET mode and a Mixed mode combining the DET and MET modes (Table 1). We then demonstrated theoretical trade-offs between amperage yield and the biomass production (growth) rate. For elucidation of the metabolic strategies for sustaining free NADH flux for electricity generation in the MET and Mixed modes, we developed a method, **F**lux variability **A**nalysis with **T**arget flux **Mi**nimization (FATMIN), to identify the enzymatic mechanisms that could be used by the cell to regenerate NADH at a high rate, subject to the network stoichiometry and substrate uptake. Finally, we analysed the effect of varying substrate uptake rate on the growth rate and the amperage output in the three modes.

**Table 1:** The metabolism and electron transfer types investigated in the present metabolic modelling

**Organism**	**Transfer type**	**Electron source (Terminal bacterial electron shuttle)**	**Metabolic type**
*G.sulfurreducens*	DET (Membrane-driven)	c-type cytochrome	Anaerobic & Heterotrophic
	MET (Mediator-driven)	NADH	
	The Mixed mode (Membrane-driven and Mediator-driven at the same time)	c-type cytochrome & NADH	

## Results and discussion

### Impact of the redox perturbation on the biomass production

**Figure 1:**
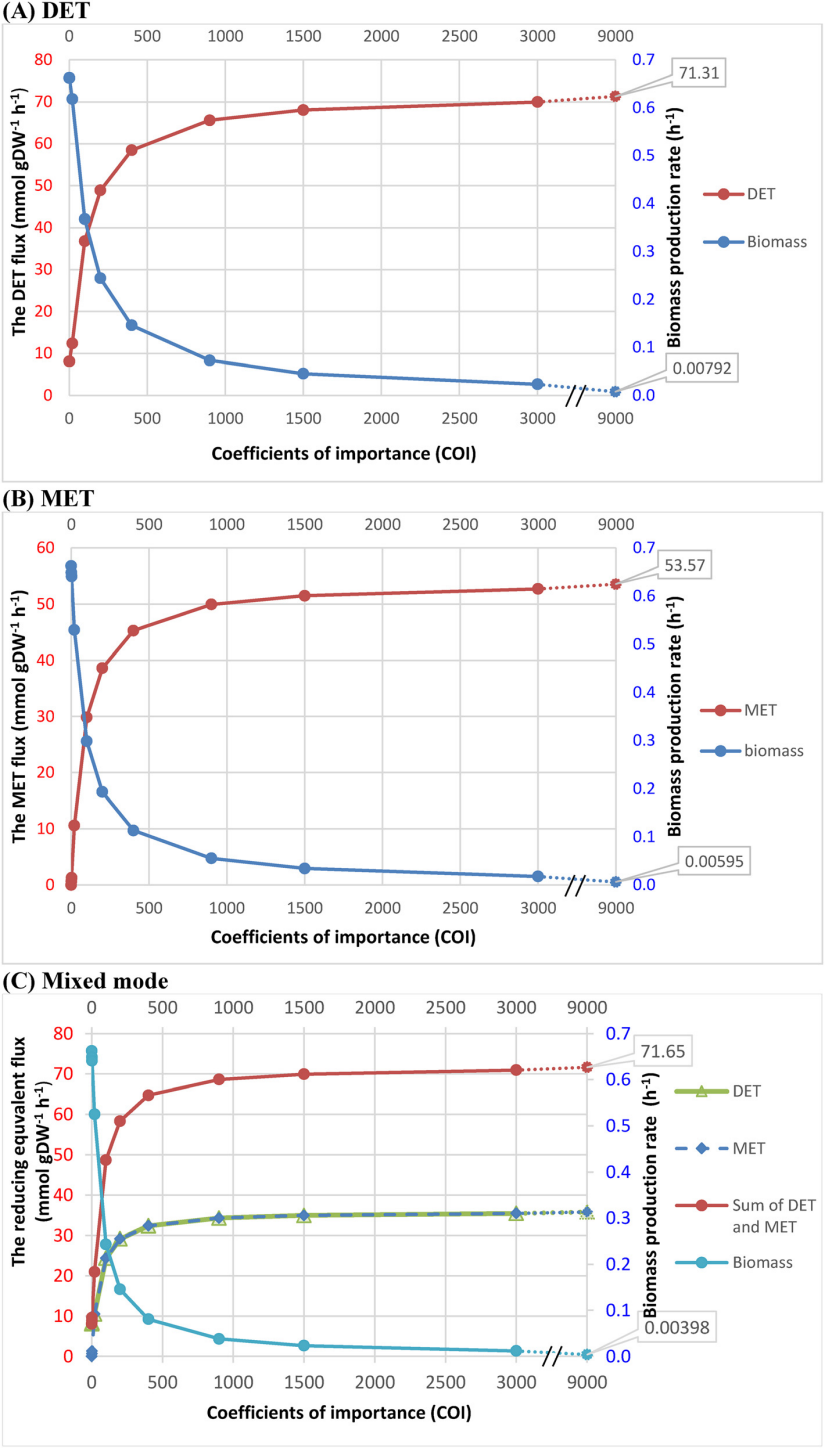
**The effect of varying COI on the biomass production and electron transfer rates.** The reducing equivalent supplying rates in the (**A**) DET, (**B**) MET or (**C**) Mixed mode, and the biomass yields in the cases of (**A**-**C**), as functions of weighting ratios of the electron transfer rate versus biomass in *G. sulfurreducens* metabolic model. A series of coefficients of importance (COI), 1,2,20, 200, 400, 900, 1500 and 3000 were applied for the electron transfer flux. The changing trends of electron transfer rates in the tree modes are compared to those of the corresponding biomass yields. COI is the stoichiometry representation used in the compound objective function. The higher the COI means the priority for associated electron transfer reaction is higher. The reducing equivalent represents NADH in the MET mode, whereas it denotes an assumed product of the reaction catalysed by cytochrome c reductase for the DET mode.

Figure 1 shows how production of reducing equivalent competes with biomass production for metabolic resources. The increase in COI drove the fluxes through electron transfer reactions in the three MFC operating modes towards their maximum allowable values and corresponding biomass formation rates to their minimum values. This reflects that at a high COI (>1000), only a small part of the energy is available to microorganisms in MFCs for their growth, as a large part is converted to electricity. The increase in COI was able to decrease the production rate of the biomass yield and increase the electron transfer much more significantly when the COI range was below 400 than above 400. This suggests that the metabolic states modelled by COI higher than 400 would be much more difficult to achieve for the microorganisms than those metabolic states with COI lower than 400. In other words, it is increasingly difficult for the cell to direct metabolic resources to sustain the increase in electricity generation. Since the COI of 400 can produce about 85%, 82% and 81% of the maximum electron transfer rates (at the COI of 9000) in the MET, DET and Mixed modes respectively, a major portion of the maximum theoretical MFC current yield should be achievable in practice for *G*. *sulfurreducens*.

There was a plateau in the electron transfer rates when the COI was higher than around 1000, which was indicated by the observation that a large increase in COI only resulted in little change in electron transfer and biomass production rates. This indicates that the metabolic states modelled by COI higher than 1000 have captured the highly perturbed metabolic behaviours under the three electron transfer modes and they were no different from one another for studying the metabolic basis of these modes.

The COI can be as large as 9000, because a large portion of this number is contributed by the high molecular weight of the biomass of the microorganisms. The coefficient for each component in the biomass equation is not a pure stoichiometric coefficient, which is different from other reactions in the model. These coefficients can convert the unit of the resulting flux through the biomass equation from mmol/gDW/h to g/gDW/h (h^-1^). An introductory exposition of the formulation of the biomass equation can be found in Feist *et al.* 2010 [17].

The present modelling allowed free selection of acetate uptake rate in a proper range (0–18 mmol/gDW/h) to maximize the objectives, i.e., biomass production rate, and NADH or c-type cytochrome involved electron transfer rates. Since acetate is the main source for producing biomass, NADH and c-type cytochrome, the acetate uptake rate stayed at the maximum (18 mmol/gDW/h) in all the simulation results. This suggests that the increased fluxes through the NADH and reduced c-type cytochrome regenerating reactions, accompanied with the raised COI, did not result from the increased availability of acetate but were associated with the redirection of electron flux for biomass formation towards mediators in the MET mode or the electrode in the DET mode.

In the Mixed mode, the total electron flux available for current production was the sum of the fluxes through the c-type cytochrome dependent and NADH-targeted pathways, which are individually exploited by the DET and MET modes respectively. The COIs influenced the combined production of the NADH and the reduced form of the c-type cytochrome in the same way as observed for the cases of MET and DET modes. Since COIs were equally assigned for DET and MET, the two electron transfer rates were nearly the same. Equal weighting may not necessarily represent actual physiological priorities, but is appropriate to evaluate how DET and MET mutually influence each other. Figure 1 shows that the Mixed mode did not produce significantly higher electron flux than either MET or DET alone. This result suggests that the DET competed with the MET for the metabolic resources.

**Figure 2:**
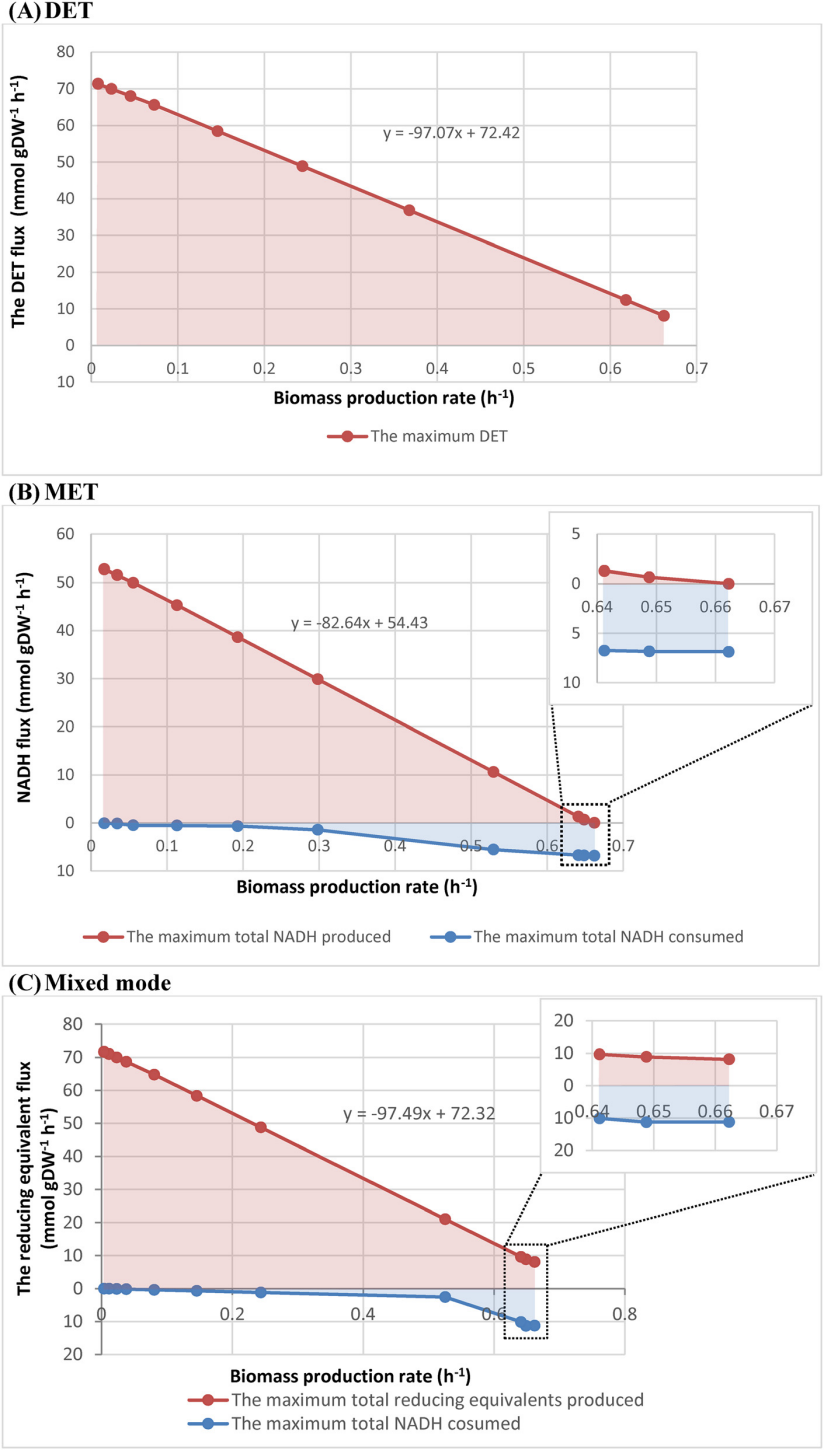
**The relationship of the biomass production and electron transfer rates.** The reducing equivalent supplying rates in the (**A**) DET, (**B**) MET and (**C**) Mixed modes, and the NADH consumption rate for cellular use in the (**B**) MET and (**C**) Mixed modes, as functions of biomass yield. The line represents the maximal electron transfer rates and biomass production rate, while any point within the pink area represents all allowable electron transfer rates and biomass production rates. The blue area represents the total NADH-consuming flux for normal cellular function. The distance between the two lines across the pink and blue areas represents the total available NADH flux in the cell at a metabolic state related to a specific biomass production rate; inset, enlargement of boxed area. The reducing equivalent represents NADH in the MET mode, whereas it denotes an assumed product of the reaction catalysed by cytochrome c reductase for the DET mode.

Figure 2 shows how reduction equivalent is allocated to internal use (i.e. maintenance of the cell metabolism) and supply to the external circuit, for various cell growth rates.

Generally, the increase in the electron transfer rates is linearly correlated with a decrease in biomass yield in the three operation modes. For DET mode (Figure 2(A)), the optimal metabolic state that resulted in the maximum growth rate of 0.6622 h^-1^ necessitates a flux of 8.092 mmol/gDW/h through reaction CYOR1m. This indicates that disposal of some electrons to exogenous Fe(III) can benefit the growth of *G*.*sulfurreducens*. When the COI associated with CYOR1m increased to 3000, which resulted in a highly perturbed metabolic state corresponding to around the upper limit of current output, the capability of the metabolic network for the electron transfer in DET mode increased by 8.65 fold (765%) to 69.69 mmol/gdW/h (not specified on the plot) compared with the optimal state.

Unlike the DET mode, which relies on one single enzymatic contribution (c-type cytochrome involved reaction), there were 51 internal reactions with potential to generate or consume NADH for the MET mode in the network model. Sum of the fluxes of all NADH producing reactions gives the total NADH production rate at a particular metabolic state, whereas sum of the fluxes of all NADH consuming reactions is the total NADH consumption rate for maintenance. The net yield of NADH reactions is the total NADH production rate available for MFCs (i.e., NADH_mfc).

For MET mode (Figure 2(B)), the total NADH production rate (sum of the fluxes of the NADH_mfc and NADH_consumed) increased from 6.865 mmol/gDW/h at the optimal growth rate of 0.6622 h^-1^ to 52.80 mmol/gDW/h at the growth rate of 0.01757 h^-1^ (COI =3000). In the metabolic state simulated by COI of 3000, the metabolism was capable of increasing the NADH_mfc yield by up to 7.5 fold (653%) compared with the base state optimised for growth. The increase in the flux of NADH_mfc was predicted to result from a combination of two mechanisms, i.e., increasing the NADH production and decreasing the NADH needed for biomass production and maintenance.

In addition, the rise in NADH regeneration rate by increasing COI accidentally activated some NADH-consuming futile cycles, leading to very high flux values (larger than 100 mmol/gdW/h) through some NADH-consuming reactions. Increasing flux through these futile cycle reactions did not change the growth rate and the net NADH production rate. Since the total NADH flux for normal cellular function was taken as the sum of all NADH-consuming fluxes, the contained futile values could make the calculated total NADH flux much higher than the actual ones. Because the task to eliminate all futile cycles in the whole network diverges from the purpose of the present study, we did not carry out the analysis of all futile pathways using methods such as elementary mode or extreme pathway, which can only be performed on a small-scale network [18].

Instead, in an attempt to eliminate flux contributions of those futile cycle reactions to the total NADH consumption rate associated with growth and non-growth maintenance, several sub-sets of constraints were used for the simulations with different COI according to a simple method (Additional file 1). This was used instead of the relatively complex pipeline in the FATMIN algorithm, because the present section simply uses a single FBA rather than elucidation of the detailed flux range of individual reactions for which FATMIN was developed.

Unlike DET and MET modes, the Mixed mode relied on the metabolic capability for concurrent regeneration of NADH and reduced c-type cytochrome. The analysis showed that the metabolism could increase the regeneration capability of the two metabolites by 3.7 fold (267.1%), compared with its base state, to 71 mmol/gDW/h (COI of 3000). The increase was attributed to a combination of three mechanisms, i.e., increasing the NADH and reduced c-type cytochrome regeneration rate and decreasing the NADH needed for biomass production and maintenance.

The mechanisms by which the NADH is additionally produced for current output are elucidated in the next section.

### Metabolic strategies for sustaining a high flux of NADH under the MET and Mixed modes

Since the further increase in COI can barely increase the electron transfer rates when the COI was above 3000, we chose the metabolic states modelled with COI of 3000 as the reference states to investigate the metabolic behaviours underlying the high electron transfer rates in the MET and Mixed modes. In fact, for identifying the metabolic behaviours under high electron transfer rates, it does not matter whether the metabolism is extremely perturbed (such as the metabolic state modelled by COI of 3000 or higher) or just a little bit less extremely perturbed (COI of 1000), as these metabolic states were all situated on the plateau of the lines (Figure 1).

#### The MET mode

When the mediator deprives NADH of electrons forming NAD^+^, the NAD^+^/NADH ratio in the metabolism is changed and consequently the cell has to adjust its metabolism to regenerate NADH from NAD^+^ in order to maintain a proper redox balance for survival. To elucidate the metabolic adjustments induced by heavy NADH loss (COI=3000), the range of variability of intracellular fluxes were calculated. The results show that although many reactions in the network can produce NADH, under heavy NADH demand, only a few reactions can sustain large NADH drain, while other NADH producing reactions become deactivated to leave the metabolic resource for the highly efficient NADH producers.

In total, ten out of fifty-one NADH involved reactions were identified as capable of regenerating NADH at a high rate under the redox perturbation (Table 2). Among the ten reactions, one reaction (AKGD) (EC 2.3.1.61) had a potential to produce NADH at up to a flux rate of 17.73 mmol/gDW/h. Each of the other nine reactions possessed capability to solely supply almost all of the 52.80 mmol/gDW/h, which was the maximum total NADH flux that could be achieved for the metabolism of *G*. *sulfurreducens* at COI of 3000. Therefore, the probed maximum NADH production rate could result from any combination of lesser fluxes of the nine reactions that made up that value. This indicates that the number of optimal solutions will be unlimited. The non-uniqueness of the obtained solution corresponds to the alternative optima in the linear optimization and the existence of alternative pathways that result in equivalent mutant phenotypes regarding the required metabolic adjustment.

**Table 2:** The identified reactions that contribute significantly to the predicted maximum NADH production rate

**Reaction ID**	**NADH flux (mmol/gDW/h)**		**Enzyme**	**EC. No.**	**Reaction**	**Subsystems**
	**Min**	**Max**				
AKGD	0	17.73	2-oxoglutarate dehydrogenase	2.3.1.61	[c] : akg + coa + nad --> co2 + nadh + succoa	Central Metabolism
ALAD_L	0	52.77	L-alanine dehydrogenase	1.4.1.1	[c] : ala-L + h2o + nad --> h + nadh + nh4 + pyr	Amino Acid Metabolism
GLUDx	0	52.72	glutamate dehydrogenase (NAD)	1.4.1.3	[c] : glu-L + h2o + nad <==> akg + h + nadh + nh4	Amino Acid Metabolism
GSADH	0	52.77	L-glutamate 5-semialdehyde dehydrogenase	1.2.1.38	[c] : glu5sa + h2o + nad --> glu-L + (2) h + nadh	Amino Acid Metabolism
HDMAT7	-0.00007	52.77	Hexadecanoyl-[acyl-carrier protein]:malonyl-CoA C-acyltransferase	1.3.1.9	[c] : nad + palmACP <==> h + hdeACP + nadh	Fatty Acid Synthesis
ME1x	0	52.77	malic enzyme (NAD)	1.1.1.37	[c] : mal-L + nad --> co2 + nadh + pyr	Central Metabolism
P5CD	0	52.77	1-pyrroline-5-carboxylate dehydrogenase	1.5.1.12	[c] : 1pyr5c + (2) h2o + nad --> glu-L + h + nadh	Amino Acid Metabolism
PDH	0	52.77	pyruvate dehydrogenase	1.2.4.1	[c] : coa + nad + pyr --> accoa + co2 + nadh	Central Metabolism
PGCD	0.0196	52.79	phosphoglycerate dehydrogenase	1.1.1.95	[c] : 3pg + nad --> 3php + h + nadh	Amino Acid Metabolism
TDMAT6	-0.00404	52.77	Tetradecanoyl-[acyl-carrier protein]:malonyl-CoA C-acyltransferase	1.3.1.9	[c] : myrsACP + nad <==> h + nadh + tdeACP	Fatty Acid Synthesis

(The reaction and metabolite abbreviations are listed in the Additional file 2).

Among the nine reactions, three reactions (i.e., AKGD, ME1x and PDH) were part of the central metabolism pathway (TCA cycle). Four reactions (i.e., ALAD_L, GLUDx, GSADH and PGCD) were related to the amino acid metabolism. Another two reactions (i.e., TDMAT6 and HDMAT7) were located in the fatty acid metabolism. Therefore, this result extends the previous notion that the TCA cycle is the main source of NADH for *G*. *sulfurreducens* when acetate is fed as the electron donor [19].

Except the nine reactions, all other NADH involved reactions were found to have very low or no variability (v_i min_/v_i max_>0.99) under high current output. The rigid variability may be attributed to the fact that NADH is a highly connected metabolite ensuring a degree of network connectivity sufficient to limit the availability of alternative flux distributions because of cascading effects on other metabolites.

The analysis presented does not fully specify the solution space. The Max/Min limits in Table 2 describes a rectangular cuboid in 10 dimensions, that encloses the feasible flux points but would in general still contain flux value combinations that are not feasible even if the values do add up to the 52.80 mmol/gDW/h value that was determined as the combined maximum. A full specification requires determination of all vertices of the solution space. Elucidation of these vertices is the task targeted by techniques such as elementary mode and extreme pathway analysis, but applications of these tools are restricted to small- and medium-scale networks of limited connectivity [20] and not suitable for the network size of *G*. *sulfurreducens* used here.

#### The Mixed mode

Similar to the case of the MET, a continuous range of solutions for achieving the high electron transfer rate in the Mixed mode involving a combination of six reactions for NADH regeneration plus one c-type cytochrome dependent DET reaction (Table 3) was obtained. It is shown that the electrons leaking from the site of c-type cytochrome influenced the possible cellular strategies to supply high NADH flux. The metabolic strategies probed for the Mixed and MET modes shared one common reaction, AKGD (EC: 2.3.1.61), to sustain high NADH flux. Nonetheless, the other eight reactions that could regenerate NADH at high rates in the MET mode were not active in the Mixed mode, which chose two new reactions, ALDD2x (EC: 1.2.1.3) and GCCc (EC: 1.8.1.4) located in the amino acid metabolism as the NADH suppliers. Besides, in the Mixed mode, another three reactions were identified capable of consuming NADH at high rates. These NADH consuming reactions were deregulated to balance the excess NADH produced by the identified three producing reactions, making up a net sum flux of NADH of 70.96 mmol/gDW/h (obtained when COI=3000). Since a range of reactions (nine for MET and three for Mixed mode) in different biological sub-systems could be targeted as the efficient NADH suppliers, NADH-dependent electricity generation should be more robust against momentary shifts in environmental conditions than the pure DET mode.

**Table 3:** The identified reactions that contribute significantly to the predicted maximum NADH production rate

**Reaction ID**	**NADH flux (mmol/gDW/h)**		**Enzyme**	**EC. No.**	**Reaction**	**Subsystems**
	**Min**	**Max**				
***NADH producing reactions***						
AKGD	0	35.48	2-oxoglutarate dehydrogenase	2.3.1.61	[c] : akg + coa + nad --> co2 + nadh + succoa	Central Metabolism
ALDD2x	8.855	35.32	aldehyde dehydrogenase (acetaldehyde, NAD)	1.2.1.3	[c] : acald + h2o + nad --> ac + (2) h + nadh	Amino Acid Metabolism
GCCc	4.427	17.66	part of glycine-cleavage complex	1.8.1.4	[c] : dhlpro + nad --> h + lpro + nadh	Amino Acid Metabolism
***NADH consuming reactions***						
GAPD	-52.96	-0.04026	glyceraldehyde-3-phosphate dehydrogenase (NAD)	1.2.1.12	[c] : g3p + nad + pi <==> 13dpg + h + nadh	Central Metabolism
GLUSx	-52.92	0	glutamate synthase (NADH2)	1.4.1.14	[c] : akg + gln-L + h + nadh --> (2) glu-L + nad	Amino Acid Metabolism
NADH5	-10.58	0	NADH dehydrogenase (Menaquinone 7 & 2 protons)	1.6.5.3	(3) h[c] + mqn7[c] + nadh[c] --> (2) h[e] + mql7[c] + nad[c]	Energy Metabolism
***DET***						
CYOR1m	35.48	35.48	cytochrome-c reductase (menaquinol 7: 1 protons)	1.7.2.2	(2) ficytcc[c] + mql7[c] --> (2) focytcc[c] + h[e] + h[c] + mqn7[c]	Energy Metabolism

(The reaction and metabolite abbreviations are listed in the Additional file 2).

In the case of MET, NADH production was increased by directing the electrons towards the reactions in the Krebs cycle and fatty acid biosynthesis, so fewer electrons are available for the downstream pathway of oxidative phosphorylation, which is associated with DET, to produce energy for the cell maintenance and growth. This accounts for the phenomenon shown here that MET and DET competed with each other for electron resources in the Mixed mode and a combination of both did not double the current output of MET or DET alone.

**Figure 3:**
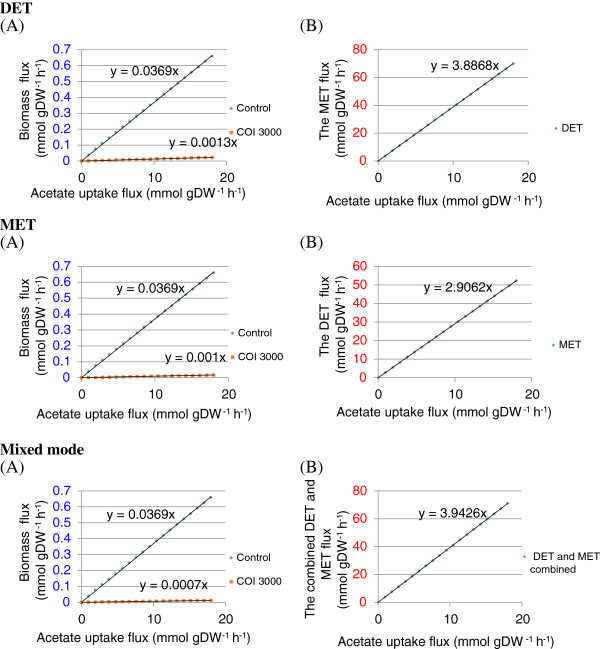
**The effect of varying glucose uptake rate on the biomass and reducing equivalent production rates. (A**) The effect of varying acetate uptake rate on the biomass growth rates when biomass production is set as biological priority for the organisms (use of only biomass maximization as objective function) and when the electron transfer in the three modes is set as the priority (use of a COI of 1 for biomass production and a COI of 3000 for the reducing equivalent production in the objective function); (**B**) The effect of varying acetate uptake rate on reducing equivalent production rate flux when a COI of 3000 is used for the reducing equivalent production and a COI of 1 for biomass production.

To demonstrate how nutrient uptake is channelled to biomass growth and current yield respectively, Figure 3 compares the corresponding fluxes at different uptake rates.

### Effect of varying acetate uptake rate on the predicted biomass and reducing equivalent production rates

The biomass production rates or the electron transfer rates were all linearly proportional to the acetate uptake rate in the three operation modes. This suggests that acetate uptake rate is the major limiting factor for both biomass and current yields. In addition, as seen from the different Y axis scales (i.e., the slopes of the trend lines), the priority set for electricity generation in the three modes lead to the suppression of biomass production and current production was much less acetate-costly than the biomass production.

The Mixed mode (slope=3.9426) can achieve higher efficiency of converting substrate into electricity in MFCs than the DET (slope=3.8868) and MET (slope=2.9062) modes. The high conversion efficiency reached by the Mixed mode is ascribed to the fact that two by-products, reduced c-type cytochrome and NADH, were both acceptable for electricity generation, leaving more freedom for the metabolism to adjust to maximize the objective than only one byproduct, such as in the either case of DET or MET.

**Figure 4:**
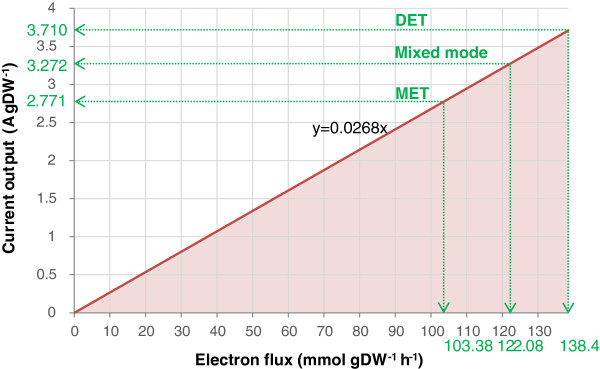
**The current output (A/g) as a function of electron flux.** The dark red line denotes the maximal current outputs and NADH_mfc production rate, while the area represents all allowable current outputs and electron production rates. The round dotted arrow line indicates the maximal current output and corresponding electron production rate when the growth rate is set to 5% of the predicted maximum growth rate (0.033 h^-1^).

Finally, the three operation modes are compared for their theoretically maximum current output in Figure 4 and Table 4.

**Table 4:** Comparison of predicted amperages and power outputs of the three modes

	**Conditions**	**Biomass growth rate (h**^**-1**^**)**	**Electron (mmol gDW**^**-1 **^**h**^**-1**^**)**	**Amperage (A gDW**^**-1**^**)**	**Coulombic efficiency (CE%)**	**Theoretical limit of the power output (W gDW**^**-1**^**)**
MET	The growth rate achieved in the acetate limiting condition)	0.06	98.94	2.652	68.71%	2.201
DET			133.2	3.570	92.50%	0.9139
Mixed mode			116.8	3.131	81.13%	2.599
MET	5% of the maximum growth rate	0.033	103.4	2.771	71.79%	2.300
DET			138.4	3.710	96.12%	0.950
Mixed mode			122.1	3.272	84.78%	2.716

Note: c-type cytochrome c and NADH have different standard potentials. The power density for the Mixed mode was calculated by sum of the power contributions from the DET and the MET, i.e., the current output of DET and MET were multiplied with their appropriate voltages respectively.

### Comparison of amperage output of the three operations modes

A growth rate of 0.06 h^-1^, which is achievable in the experiment [21], was also chosen as a reference value in addition to the 5% optimal growth rate. Among the three tested electron transfer modes, DET delivered the highest maximum limits of the current output, whereas MET achieved the lowest maximum limit. This could be attributed to the fact that *G*. *sulfurreducens* can conserve energy for cellular growth during the process of disposing electrons to exogenous electron acceptor [22] and consequently less additional energy resources (i.e., acetate) are required for DET mode to achieve the same level of current output as the MET. Therefore, these organisms can gain an energetic advantage by growth on or near the electrode. Nonetheless, despite its higher current output, the maximum power output of the DET mode was lower than those achievable by the other two modes, due to the low standard potential of c-type chromosome used by the DET mode.

For the MET, mediators are needed to extract the redox potential out of cells and thus the cells that are located far away from electrode surface can still contribute to the current production. On the other hand, the DET depends on the 2–3 μm long pili of *G*. *sulfurreducens* to deliver the electron from the outer membrane to the anode [23,24]. Consequently, compared with the MET, the DET requires a much closer distance between the cell and the anode surface, and thus the actual current output will be restricted by the number of electrochemically active cells that near the surface area of the electrode. This may account for the observations that the DET based systems usually deliver quite low current [6].

The result shows that the maximum output of the Mixed mode was a little bit higher than MET. However, based on this, it cannot be concluded that Mixed mode is more competitive than MET. In effect, the Mixed mode may require independent circuits to collect the power output from the DET and MET concurrently. Furthermore, the Mixed mode requires involvement of mediators, large surface contact between the cells and special electrode design that can accept electrons from the two reducing agents with different potentials, which creates more demands for the engineering design and has impacts on power density and the long-term stability of the MFCs. The extra complication probably does not justify the design effort needed for mixed mode operation.

The DET and Mixed mode involved higher coulombic efficiencies than MET mode, which implies that more of electron flows were directed to current production rather than cellular synthesis. In a previous MFC review article, the MET system was suggested to produce higher current and power densities, but have lower coulombic efficiency than the DET [6]. This is because part of the electrons from the substrate are used by the cell to form some electrochemically inactive side products [6].

The present study computed the theoretical maximum CE% achievable for the DET as 79.34% based on the stoichiometry in the network, indicating the acetate-fed MFCs using *Geobacter* species can effectively oxidize acetate with electron transfer to electrodes. However, the computed value is still much lower than the values reported by several previous studies, which claimed that *G*. *sulfurreducens* in MFCs could convert acetate to current with CEs of 89% [25] and above 90% [26,27]. And some studies assumed a CE of 100% for the DET via c-type cytochromes [28,29].

The reasons for such a discrepancy between the computed CE in this study and previous reported ones are speculated to be as follows: 1) The acetate consumed by the cell was calculated by measuring the change of concentration of acetate in the culture media. The measured point is always far away from the cells. A gradient of acetate concentration exists in the distance between the measured point and the cell surface, i.e., the acetate concentration is lower at the cell surface and much higher at the point where the measurement takes place, because starting with a homogenous acetate concentration, after MFC operation the concentration will drop more at the cell surface than at the measurement point. Thus the measured acetate uptake rate is lower than the actual uptake rate. According to the formula: $\mathrm{CE}\%=\frac{\mathrm{Acetate}\;\mathrm{used}\;\mathrm{for}\;\mathrm{current}\;\mathrm{production}}{\mathrm{Total}\;\mathrm{acetate}\;\mathrm{consumed}\;\mathrm{by}\;\mathrm{the}\;\mathrm{cell}}$, a underestimated total acetate uptake rate could increase the resulting CE%. 2) The whole experiment period did not last long enough for the microbes to form a steady state. 3) Microbes can consume other nutrients in the media or reserved in the cells, such as other dead cells, for electron production, but this factor was not taken into account for calculating the total substrate consumed by the cell.

Microorganisms have a strong propensity to aggregate and proliferate into biofilm on the solid surfaces [30]. A previous MFC study reported a current output of 1.4 A/m^2^ and a power density of 0.5 W/m^2^ by a biofilm of wild-type *G*. *sulfurreducens*[27]. The cell protein density of the biofilm was measured as 0.571 g protein/m^2^[27]. With an assumed protein content of 46% of cell dry weight [31], the cell density of the biofilm is estimated to be 1.241 g/m^2^ and the previously reported current flow (1.4 A/m^2^) and power density (0.5 W/m^2^) can be converted into 1.128 A/gDW and 0.4028 W/gDW respectively. Clearly, these values are much lower than the computed ones (3.710 A/gDW and 0.95 W/gDW) for the DET mode in the present study. The difference between the *in silico* predicted maximum electric outputs and observed ones may be attributed to the fact that some electrons and potential would be lost during the electron transfer between the biofilm and the electrode, and consequently the actual current and power density will be lower than these computed values. Understanding and optimizing the micro-electrode interaction is another challenge to overcome for the MFC research.

It is commonly observed that mixed cultures usually produce much higher current densities at high coulombic efficiency compared with pure cultures, due to many advantageous features arising from the mutualism among microbial diversity [12,32]. However, by improving the engineering design, the MFC based on *G*.*sulfurreducens* can produce a power density similar to those reported with various mixed cultures while maintaining high coulombic efficiency [26].

## Conclusions

Analysis of the metabolic flux models predicted that *G*. *sulfurreducens* in the DET mode achieved the highest current output (3.710 A/gDW), followed by the Mixed mode (3.272 A/gDW), and MET the lowest (2.771 A/gDW). In the DET mode, the enzymatic activity of one reaction determined the current generation in MFCs. On the contrary, in the MET mode, the reducing molecule, NADH, could be efficiently regenerated and supplied from ten identified reactions, while in the Mixed mode the number of the possible reactions supplying NADH reduced to three. The decreased reaction number indicates that the DET and MET compete with each other for the metabolic resources. The increases in the electron transfer rates in the three modes were linearly correlated with the drop in growth rate, which was, however, achieved by the increasingly rising metabolic ‘cost’ (modelled by COI).

The multiplicity of reactions capable of individually supplying the maximal current output indicates a range of feasible solutions, rather than just a single idealised metabolic state. This leaves room for good performance to be obtained from different cellular phenotypes and suggests robustness to changes in environmental or metabolic conditions.

While this work identified the maximum capability of *G*. *sulfurreducens* for current production, this leaves open the question of practical strategies to achieve this potential. One possibility may be a metabolic engineering strategy such as adaptive evolution. In this way, if the right selection pressure is applied, there is a continuous selection for beneficial mutations that can attain the high predicted current output. As an alternative approach, gene (reaction) deletion can also be a fast way to generate beneficial mutant strains, facilitated with the reaction knockout identifying algorithms such as OptKnock [33-35] and OptGene [36,37].

## Methods

### Flux variability analysis with target flux minimization (FATMIN)

#### Background

FBA relies on linear programming to find the optimal solution for reaction fluxes, which maximizes or minimizes a given objective function. However, the identified solution may not be unique and an infinite number of different flux vectors may exist producing an identical optimal objective value. In the context of metabolic models, these flux vectors are called alternate optimal solutions (AOS) or equivalent phenotypic states [38-41]. Previous methods for the determination of AOS include 1) vertex enumeration such as extreme pathway or elementary mode analysis [40,42,43], and 2) flux variability analysis (FVA) [44]. However, the computation of extreme pathways and elementary modes are too time-consuming and sometimes impossible, even for a medium-sized network model (up to one hundred reactions), and FVA results may contain large futile values due to the presence of loops in the network.

**Figure 5:**
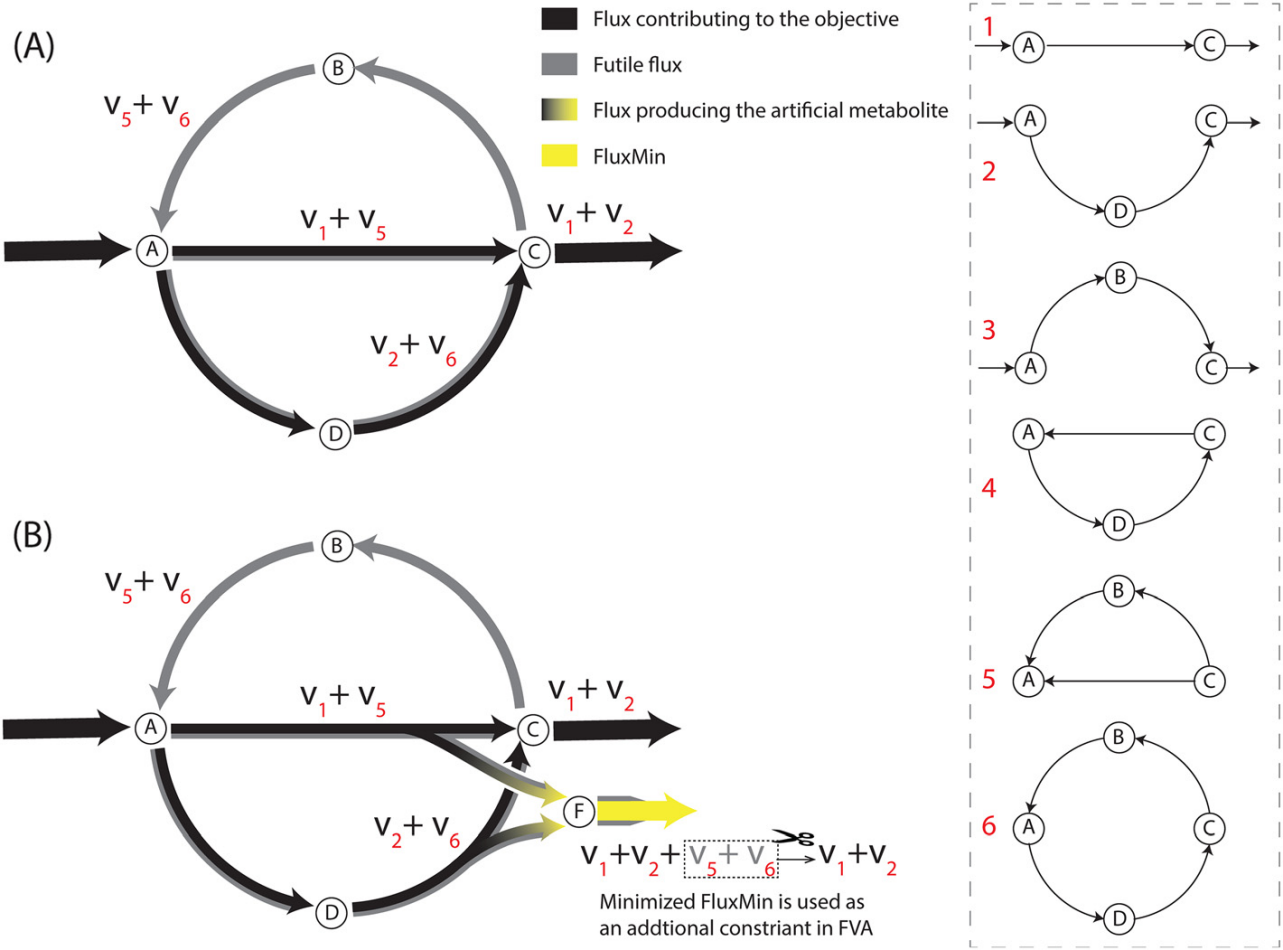
**The schematic diagram of elimination of the futile cycle in an imaginary network by the FATMIN.** (**A**) Flux distribution at steady state without employment of the FATMIN; (**B**) The minimization in the FATMIN to identify another constraint that can remove the futile values associated with cycles 5 and 6. The inset shows alternate path through the network, with numbers used as flux indices in the main figure. Path 3 and 4 carry no flux with reaction directions as shown, but are examples of paths that become active if reactions are made reversible.

In constraint-based modelling, the constraints mainly used are mass balance and metabolite uptake rates. However, thermodynamic limitations associated with the reactions are difficult to implement and commonly overlooked. Without thermodynamic constraints, non-physical fluxes can be computed for some metabolic reactions if they form an internal cycle (Figure 5(A)). Such a cycle of reactions violate a "loop law" that is analogous to Kirchhoff’s second law for electrical circuits, as discussed previously by Beard *et al.*[45].

In practical FBA calculations, the presence of a futile loop is often signalled by implausibly high flux values for some reactions. It is common practice to set an artificial upper limit of e.g. 10000 on all fluxes, simply to avoid numerical problems in the optimization algorithm. Fluxes of loop reactions are often observed to reach values of this magnitude, but simple strategies such as blocking these reactions by constraining their fluxes to zero only work in the simplest cases.

One reason for this is that individual loop reactions may simultaneously also participate in productive (non-futile) fluxes carried by active pathways. In this case flux values along the loop may all be high, but with different values, because the observed fluxes result from superimposing productive and futile fluxes. This has to be disentangled without affecting productive fluxes. This problem is particularly common when loops involve a currency metabolite such as NADH.

Another reason is that as loop fluxes do not contribute to the objective value, it is conceivable that some such fluxes may have another value, even zero, in the particular flux distribution that is selected from the solution space by the optimization algorithm.

More elaborate strategies that have been proposed to remove these thermodynamically infeasible futile cycles, include known flux directionality [46], energy-balance equations [45], known [47-52] or predicted [53] thermodynamic parameters, and nonlinear constraints to eliminate flux distributions that utilize reactions which cannot be thermodynamically feasible under physiological conditions [54]. But these thermodynamic constraints are difficult to obtain and nonlinear constraints can make application to large-scale systems computationally challenging [48]. Therefore, the listed methods above cannot be applied on genome scale systems [48].

Alternatively, loops can be removed by minimizing network flux [47,55-57]. The application of flux minimization can produce a most likely stationary flux distribution. The notion behind this method is that the flux through any reaction of a metabolic network requires some ‘effort’ and a metabolic network is inclined to fulfil the same biological objective, such as growth, with minimum metabolic effort [47]. A drawback of this approach is that the use of the minimum sum of all fluxes in the network as an auxiliary criterion in FBA by previous methods, can forestall variability within optimal flux distribution.

Below, we present a new method based on FVA and FBA to determine AOS and efficiently eliminate flux loops in large metabolic networks.

### The FATMIN algorithm

The basis of FATMIN is FVA, which is used to probe the feasible flux ranges of desired reactions, such as NADH involved reactions in the present study. Importantly, the method can constrain the pointlessly high fluxes values of the loop reactions to a biologically meaningful value. It is intended to deal with such loops involving one or more *target metabolites* that are of specific interest; here this is NADH.

All reactions that produce the target metabolite and carry a flux higher than a threshold value (that is chosen to be higher than any realistic flux value) are identified as *target reactions*. To identify which portion of the high value is compulsory to achieve the value of the FBA objective function, we add an artificial metabolite to each target reaction, and add another reaction (e.g., reaction ID: FluxMin) that only comprises the artificial metabolite. FluxMin acts as a drain reaction for the artificial metabolite, and its flux is the sum of all production fluxes. Minimizing this flux while maintaining the objective value, in effect eliminates futile fluxes.

The idea is demonstrated in Figure 5(A). Metabolite C is taken as the target metabolite that this simple network produces. The network contains two alternative routes (1 and 2) for producing C and two cycles (5 and 6). All reactions are irreversible, so with paths through the network chosen as indicated, all flux values are nonnegative. A new metabolite labelled “F” is added to both reactions that produce C, as well as the associated drain reaction, to give the augmented network in Figure 5(B). Balancing fluxes at all nodes of the augmented network shows that although the net production of C (made up of the contributions v_1_ and v_2_) is independent of the futile fluxes, the flux of the added reaction FluxMin is v_1_ + v_2_ + v_5_ + v_6_ . Since the value of v_1_ + v_2_ remains fixed for a chosen objective value and negative values for v_5_ and v_6_ are not allowed, minimization of FluxMin reduces both of the cyclic fluxes to zero. The resulting minimal value is subsequently used as a constraint on FluxMin in a repeated FVA calculation for the augmented network.

Although the explanation above referred to fluxes along individual pathways – in effect, elementary modes – the strength of the FATMIN method is that these do not in fact have to be identified to apply the method. Simply adding FluxMin to the network and minimizing its flux ensures that the cyclic components implicitly contained in it are eliminated.

This result relies on the fact that negative flux values are excluded. Realistic networks containing reversible reactions are usually handled in FBA by allowing positive and negative flux values. To maintain the exclusion of negative fluxes, in the FATMIN minimization step reversible reactions involving the target metabolite are conceptually split into two counter directional reactions, and the artificial metabolite only entered into the branch that produces the target metabolite. In practice, because the other branch does not contribute to FluxMin, it can be omitted. So the procedure followed is that where the FVA range of a target reaction exceeds the threshold on both negative and positive fluxes, the negative lower limit constraint is replaced by zero for finding the minimal FluxMin flux value. We stress that for the subsequent FluxMin-constrained FVA calculations the lower limit is restored to its original value.

Another point illustrated by the example is that the FATMIN strategy does not restrict the optimal solution space; e.g. all v_1_ and v_2_ value pairs allowed by the original objective value remain viable in the augmented network. Finally, we remark that the strategy described here is easily extended for multiple target metabolites by simply introducing a unique artificial metabolite and drain reaction for each.

Overall, FATMIN can be summarised as a modelling sequence FBA+ FVA +FBA +FVA, which is implemented as a computational pipeline consisting of the following steps, where we take NADH as the target metabolite:

1. Perform FBA to calculate the optimal objective Z_optimum_. Then set the obtained optimal Z_optimum_ as the constraint and perform flux variability analysis (FVA) to calculate a feasible flux range for each reaction.

2. Extract NADH reactions and calculate the feasible range of NADH flux values associated with each reaction by multiplying reaction rate with NADH stoichiometry coefficient.

3. Any reaction with an absolute value of the NADH flux boundaries higher than a threshold T (here, we chose T = 100 mmol/gDW/h) is identified as a target reaction. In such a reaction, if the absolute value of the lower limit is higher than T, we decompose the reversible reaction into two reactions in forward and backward direction respectively.

4. Extend the network by adding an artificial metabolite F as a product to each target reaction, and an F drain reaction FluxMin to the network model.

5. Use the FBA optimum (Z_optimum_) as the constraint and minimize the flux of reaction FluxMin.

6. Perform FVA on the augmented network while using the obtained minimal flux value obtained at step 5 as a constraint for the flux of FluxMin.

7. Re-calculate the numerical range of NADH flux values using the method in step 2.

This method shares some ideas with [58], in which the solution space is described in terms of three key characteristics: linealities which are the reversible (bi-directional) infinite reactions, rays which are the irreversible infinite reactions, and vertices which are the corner points of the shape formed by interception of the polyhedral cone representing the convex constraint space with the objective plane. However, there could be millions of vertices, from which one cannot identify biological significance. Therefore, FATMIN intends to remove only the rays and linealities, which matched irreversible and reversible cycles. The rays and linealities are independent of the growth medium [58]. Since FATMIN is based on FVA, the method inherits the function of the FVA in elucidating the phenomena of equivalent optima in the network. Nonetheless, unlike the results of FVA that could contain infeasible, futile values, the result of FATMIN are the feasible flux ranges for the reactions.

The drawback of FVA is that this tool only produces the outline of a rectangular cuboids or ‘box’ encompassing the polygon of the solutions, but cannot illustrate the real shape of the polygon, which indicates that FVA results cannot reflect the relationships between different reaction fluxes, e.g., how the increase in the fluxes of some reactions influence the fluxes of the other reactions. The solution ‘box’ is discussed in the case of the MET (The MET mode section of the Results and discussion).

### Assumptions for present modelling

To model the metabolisms under electricity generation, a few assumptions were made: 1) the MET mode relies on a putative ideal mediator (e.g., Neutral red [59]) that can enter cytoplasm and transfer electrons from NADH/NAD^+^ cycle to anode. 2) Only pure culture is used for electricity generation. 3) The MFC reactor is a chemostat that can provide optimum conditions to meet all the needs of microbial growth. Subsequently, in order to ensure that the cells are not limited by substrate uptake rates, the model is allowed to adjust substrate uptake rate within a proper range as necessary for optimal growth yield. The range constraints on the reactions used in the modelling are specified in the model (Additional file 3). In addition, since the carbon and energy source were limited to a proper range, there is no necessity to use a two-step optimization strategy, i.e., the first is to maximize biomass with no constraint and the second is to minimize energy usage while fixing the biomass, as described in [31,60,61].

### Modelling electrode interactions

**Figure 6:**
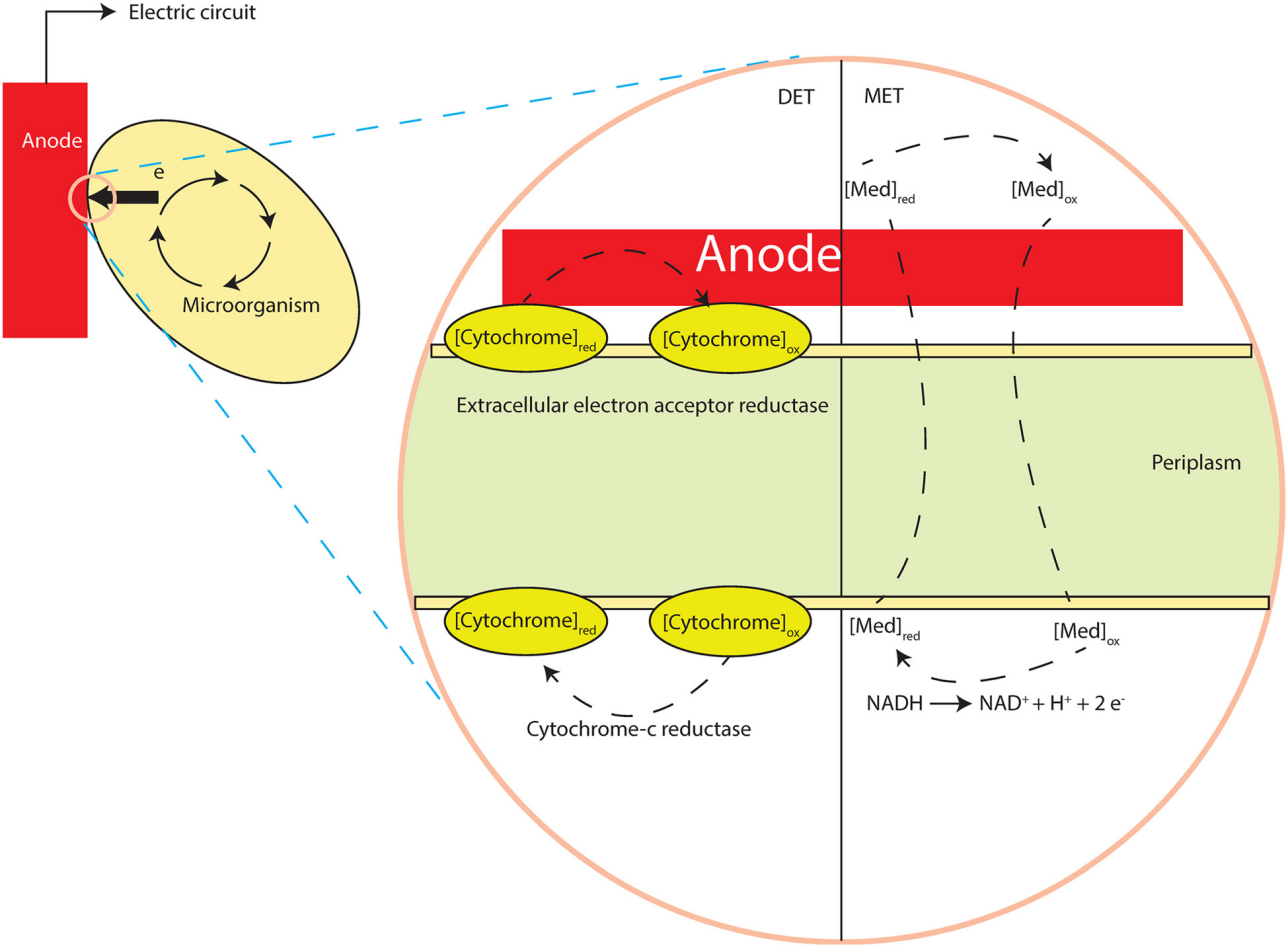
**A schematic of the anodic mechanisms in MFCs.** (i) DET, the electron transfer involving c-type cytochrome. (ii) MET, the electron transfer driven by a mediator used process; red, reduced form; ox, oxidized form. Microbes take up substrate generating carbon dioxide and protons. This process yield electrons for metabolic benefit, i.e., growth, and reduce Med_ox_ in the cytosol into Medred. Med_red_ diffuse into contact with the electrode, where Med_red_ reduce the electrode generating electrical current. The oxidized form, Med_ox_, diffuses back through anolyte for reuse by the microbes. Periplasm (only exist in gram-negative bacteria such as G. sulfurreducens).

The interactions with an electrode were captured by introducing three reactions into the model reconstruction (Table 5). These reactions represent the net reaction that takes place between the reducing equivalents and the mediators (or electrode) in MFCs. Introduction of these reactions create additional channels for electrons to escape from the metabolism and the fluxes of these reactions are subject to the mass balance rule in the FBA modelling. Because NADH and NAD are native metabolites of the microorganisms, these added equations did not lead to non-native by-product production. The reducing equivalent represents NADH in the MET mode, whereas it denotes an assumed product of the reaction catalysed by cytochrome c reductase for the DET mode. In both cases, one cycle of the electron transfer yields two electrons. The process is schematically shown in Figure 6.

**Table 5:** The added reactions for modelling the interaction of the microorganisms and the electrode in MFCs

**Operation mode of the MFC**	**Reaction ID**	**Reaction**
MET	1NADHmfc	nadh --> nadh_mfc
	2NADHmfc	nadh_mfc ->nad+h_emm
DET	3Cytcmfc	h_edm --> h_edm_out

nadh_mfc: the NADH available for MET mode of MFCs;

h_emm: the H ions as the by-product released from the reaction 2NADHmfc;

h_edm: the H ions as the by-product released from cytochrome-c reductase.

(CROR1m) catalysed reaction; h_edm_out: the extracellular form of h_edm.

Scanning previous MFC studies indicates that the electron-transfer property of mediators can be specific to each cell design and vary between cells and operational conditions even for the same reaction. This indicates that there is no such a certain mediator ‘supreme’ for all MFCs. Therefore, to avoid limiting the scope of the present study to a particular mediator and leave room for researchers to refine the electron-mediating properties of mediators, we choose to model the function of mediators, skipping their properties such as electric potential and the ability to penetrate the cellular membrane.

### Locating the target site in the DET mode

**Figure 7:**
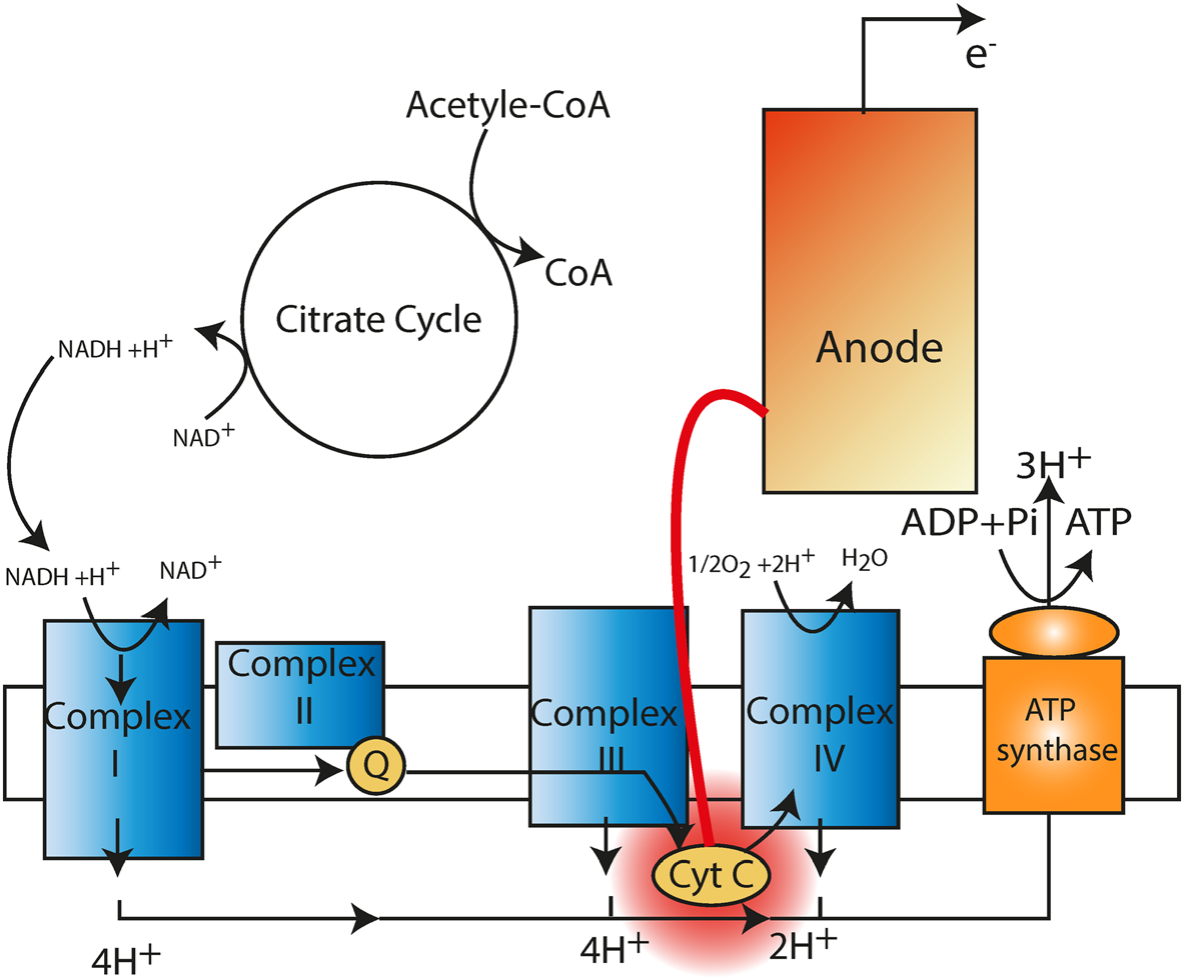
**A schematic illustration of the electron source in direct electron transfer mode.** Cyt C, Cytochrome c, which is not part of an enzyme complex and moves between complex III and IV as a freely soluble protein.

The c-type cytochrome is the electron source for the DET mode (Figure 7) [62]. In the network model, the reaction that captures the c-type cytochrome production and the direct electron transfer to the exogenous electron acceptor are retrieved and listed in Table 6.

**Table 6:** **The reactions of the extracellular electron transfer in *G. sulfurreducens***

**Reaction ID**	**Enzyme**	**Reaction**
CYOR1m	cytochrome-c reductase	(2) ficytcc[c] + mql7[c] -->(2)focytcc[c]+h_edm [e] +h[c] + mqn7[c]
FERCYT	Fe (III) Reductase	fe3[e] + focytcc[c] --> fe2[e] + ficytcc[c]

(The reaction and metabolite abbreviations were described in Sun, *et al.* (2009) [63] and also in the Additional file 2).

The reaction CYOR1m makes the model suitable for studying direct electron transfer in the case of MFC. The availability of the c-type cytochrome for extracellular electron transfer is determined by the flux of this reaction. It is assumed that one round of this reaction cycle produces two quotients of the reduced c-type cytochrome for the extracellular electron transfer reaction (FERCYT), equivalent of two quotients of electrons for the electric circuit.

### Objective equations

For modelling the maximum metabolic potential for the current production and the pertinent metabolic behaviours in the MET mode, the cells were tested for their capabilities to convert low energetic co-substrates (NAD) to their highly energetic counterparts (NADH). For the DET mode, the oxidation rate of c-type cytochrome by the exogenous electron acceptor (i.e., the anode) was maximized. For the Mixed mode, the maximization of the rates of the MET and DET were equally combined. Hence, the Mixed mode was just a special case of the MET mode with the reduced type c cytochrome as an additional electron supplier.

The present study used an optimization based framework based on a hypothesized objective function produced by a weighted combination of fluxes (Table 7). Characterizing changes to NADH yield and biomass growth rate can aid in understanding the impact of the enhanced current extraction on cellular metabolism. We used a compound objective function comprising maximizations of NADH and biomass concurrently. The weighting for each component objective is termed as the coefficient of importance (COI), which was used to define the competing priority of one objective with respect to the other one. Thus, the objective functions were defined by setting weightings on multiple reactions (i.e., a set of COIs). COI can also serve as a tool to quantify the fraction of the additive contribution of a given flux to a fitness (objective) function [35]. In this way, the metabolic ‘cost’ for achieving a desired objective was quantitatively evaluated.

**Table 7:** Formulation of objective functions for modelling perturbed metabolisms under electricity generation

MET	Biomass production + (COI_DET_) NADH_mfc
DET	Biomass production + (COI_MET_) h_edm_out
Mixed mode	Biomass production+(COI_MET_)NADH_mfc+(COI_DET_) h_edm_out

Note: NADH_mfc denotes the NADH flow that contributes to the electricity generation; h_edm_out represents the flux of the DET reaction that involves the cytochrome c reductase.

A high COI implies that the corresponding flux is preferentially maximized by the network, whereas a low COI indicates the converse (i.e., a low priority for the maximization). In the present modelling, COI can also be considered as the degree of NADH perturbation caused by the mediators of MFCs. A high COI magnitude associated with NADH is used to model NADH drain in MFCs, while the COI assigned to the biomass production by default is 1 in the network model.

The use of COI is also a way to represent regulatory mechanisms of the cell to increase the electron transfer rate in DET and MET for MFCs while trying to maximize the cellular growth rate. The mediator in MFCs deprives the cell of the electrons, and the cell metabolism reacts to the deprivation by regulating the metabolism to increase NADH production because the cell requires NADH for growth. In other words, we use the growth objective to model the normal cell metabolism. Then we introduce the COI to model the regulation process of the cell (Table 8).

**Table 8:** The principle of the bi-objective optimization in modelling regulatory mechanisms under energy extraction

**Objective one**	**←**	**Regulation**	**→**	**Objective two**
(Maximization of growth rate)		(modelled by way of COI)		(Maximization of election transfer in DET and/or MET, which mimics the metabolic perturbation caused by DET and/or MET for electricity generation)

The growth-coupled product flux ensures that the metabolic network is forced to produce the desired reaction flux while still supporting a functional state of the network for a biologically meaningful goal, i.e., to grow (to survive). The predictions of these models on growth rate have been verified by the experimental studies where the original network was published [31].

The values of the COI’s in this study should not be directly interpreted as specifications of the relative priorities of the two objective functions, because due to the different units of measurement of biomass and reduction equivalent objectives, they also contain a unit conversion factor. The value of this factor is not easily determined because it is related to the “molecular weight” of the biomass, but it is not clear what meaning to attach to one “molecule” of biomass.

### Conversion of units of flux and current

Current (in amperes) was integrated over time and converted to electrons recovered by using the following conversions: 1 C = 1 A × 1 s, 1 C = 6.24 × 10^18^ electrons, and 1 mol = 6.02 × 10^23^ electrons (Faraday's constant 96485 C/mol). Therefore, one flux unit (mmol/g/h) can be converted into A/g as follows:

$$1\;\mathrm{mmol}/g/h=\frac{1\;\mathrm{mol}}{1000g\times 3600s}\times \frac{96485C}{\mathrm{mol}}=0.0268A/g$$

### Coulombic efficiency (CE)

One of the parameters commonly used to quantify the performance of MFCs is the Coulombic efficiency (CE); the CE is defined as the ratio of electrons transferred to the anode to that in the starting substrate. For the characterization of the energy efficiency of the metabolism, the full oxidation of acetate with oxygen as the oxidant (see as follows) will be used as the reference reaction.

$$\begin{array}{ll} \text{Acetate oxidation reaction}: & CH_{3}\mathrm{COOH}\cdot +\cdot 2\cdot H_{2}O\cdot \to \cdot 2\\ & \cdot CO_{2}\cdot +\cdot 8\cdot H^{+}+\cdot 8\cdot e^{-}. \end{array}$$

Based on such stoichiometric information, 1 mol of acetate was converted into 8 mol of electrons (1 mol of acetate = 8 mol e^-^) [64].

$$\begin{array}{ll} \mathrm{CE}\%= & \frac{C_{\mathrm{output}}}{C_{\mathrm{substrate}}}\times 100\%\\ & =\frac{\mathrm{reducing}\;\mathrm{equivelent}\;\mathrm{produced}\;\left(\mathrm{mmol}/\mathrm{gDW}/h\right)}{\mathrm{acetate}\;\mathrm{uptake}\;\mathrm{rate}\;\left(\mathrm{mmol}/\mathrm{gDW}/h\right)\times 8}\% \end{array}$$

### Calculation of theoretical power outputs of the three tested modes

The calculations in the present study are only based on a flux balance viewpoint and thus do not take into account ohmic resistances, concentration polarization and kinetic constraints during a MFC operation. For the sake of simplification the calculations of cell voltage (i.e., cell potential) are based on formal potentials (at pH 7) of the involved biological and electrochemical redox processes. Therefore, this calculated Electromotive force (EMF) provides an upper limit for the cell voltage and the actual potential derived from the MFC will be lower due to various potential losses [65]:

$$\Delta E_{\mathrm{cell}}^{\circ '}=E_{\mathrm{cathode}}^{\circ '}-E_{\mathrm{anode}}^{\circ '}$$

Where $\Delta E_{\mathrm{cell}}^{\circ '}$ is the standard cell potential (aka., electromotive force); $E_{\mathrm{cathode}}^{\circ '}$ is the standard potential of cathode oxidation; $E_{\mathrm{anode}}^{\circ '}$ is the standard potential of anode reduction.

The formal potentials of the anode and cathode used for calculation of power density in the three operation modes were summarized in Table 9.

**Table 9:** The standard potential of the anodic and catholic reactions funneling electrons to the electrode (measured at pH 7)

		**Redox couple**	***E°*′ (V)**
Anode	MET	NAD^+^+H^+^+2e^-^→NADH	-0.320 [66]
	DET	Cytochrome c(Fe^3+^)+e^-^→Cytochrome c(Fe^2+^)	+0.254 [66]
Cathode		O_2_+4H^+^+4e^-^→2H_2_O	+0.51 [6,67,68]

The calculation of standard cell potential of MFC (Table 10) shows that as long as the same electron donors are used as fuel there is little that can be done in terms of discovering bacteria capable of creating stronger potentials.

**Table 10:** **The theoretical limit of standard anode potentials of MFC based on *G sulfurreducens***

**Electron transfer mode**	$E_{\mathrm{anode}}^{\circ '}$	$E_{\mathrm{cathode}}^{\circ '}$	$\Delta E_{\mathrm{cell}}^{\circ '}$
MET	-0.32	0.51	0.83
DET	0.254	0.51	0.256

### Calculation of the viable growth rate

Several modelling works assumed 1% of the maximum theoretical biomass yield as the viability threshold for computational identification of lowest growth rate [69,70]. Nevertheless, we chose 5% of the maximum theoretical biomass yield in this study since this value is more conservative and more likely to be practically viable.

### Simulating the growth of *G*. *sulfurreducens*

The flux values in the simulations were in units of mmol/gDW (dry weight)/h. The maximum acetate uptake rate was constrained to 18 mmol/gDW/h [71]. Non-growth associated ATP maintenance demand was set to 0.45 mmol/gDW/h [31,72]. The maximum Fe3+ and NH_4_^+^ uptake rates were set to 568 mmol/g/h and 0.468 mmol/g/h respectively [71]. The exchange rate of other external metabolites such as CO_2_, H_2_O, K^+^, Mg^2+^, PO_4_^3-^, and SO_4_^2-^ were allowed to freely enter and leave the network [31].

### Analysis technique

An updated metabolic network model of *G sulfurreducens* PCA strain [31,63] was used as the backbone for all *in silico* analysis. The original network model in Excel format was parsed into SBML using the converter integrated in the NetSplitter [73,74]. The growth rates and electron production were computationally determined using Flux Balance Analysis (FBA) [75,76]. Computations were performed with COBRA Toolbox [77] in MATLAB (The Math-Works Inc., Natick, MA, USA) and OptFlux [78]. FATMIN is a pipeline comprising FBA and FVA and thus can be performed with either COBRA or OptFlux according to users’ preference.

## Abbreviations

MFC: Microbial fuel cell; NAD: Nicotinamide adenine dinucleotide; NADH: Nicotinamide adenine dinucleotide (reduced form); DET: Direct electron transfer; MET: Mediated electron transfer; BG: Bromocresol green; NR: Neutral red; COI: coefficient of importance; AOS: alternate optimal solutions; FATMIN: Flux variability analysis with target flux minimization; FBA: Flux balance analysis; FVA: Flux variability analysis.

## Competing interests

The authors declare that they have no competing interests.

## Authors’ contributions

LM and WV conceived of the study, developed the method and wrote the manuscript. LM performed *in silico* modelling and modelling analysis. WV supervised the work. Both authors read and approved the final manuscript.

## Supplementary Material

Additional file 1 A simple method to eliminate encountered futile cycle reactions during FBA modelling. Click here for file

Additional file 2 Flux profiles and FATMIN results. Click here for file

Additional file 3 **SBML file of the *G sulfurreducens *network model.** Click here for file
