# Supplementary material for: Model-driven elucidation of the inherent capacity of Geobacter sulfurreducens for electricity generation
Source: J Biol Eng. 2013 May 29;7:14. doi: 10.1186/1754-1611-7-14 (PMC3673867; doi:10.1186/1754-1611-7-14)
Supplement: Additional file 1 — A simple method to eliminate encountered futile cycle reactions during FBA modelling. [file 1754-1611-7-14-S1.docx]

**A simple method to remove futile reactions encountered in the FBA modelling of the MET mode.**

It is important to eliminate the large portion of the futile values associated with the obtained reaction fluxes since these values have no biological meanings. If sum of the flux values of selected reactions are larger than 1000, there should be some reactions in futile loops (Figure S1). These large values are explicitly-encoded infinity constraints i.e. bounds on reactions represented by a large number. To reduce the large futile value to a meaningful value that is essential for the objective value, we employed a simple method to remove those encountered loops. i.e., If several futile values (i.e., >1000) exist in a number of selected reactions, we constrained the flux with the smallest absolute value to zero in order to break the loop.

| (A) | 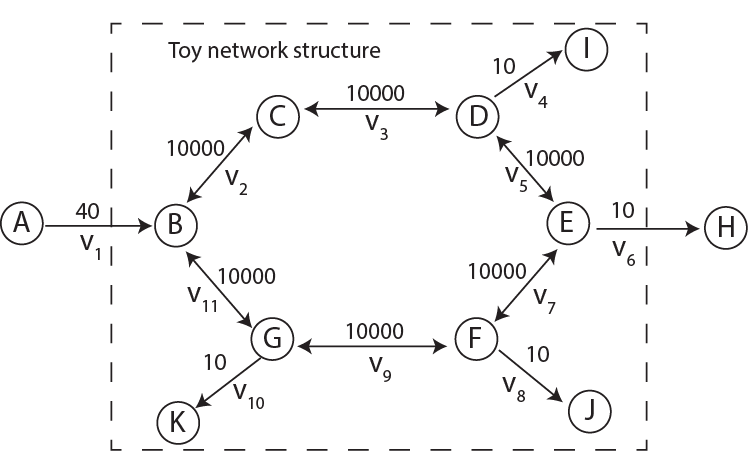 |
| --- | --- |
| (B) | 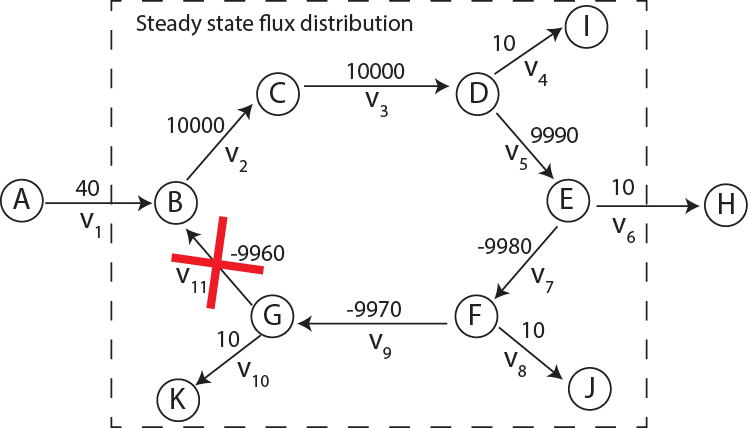 |
| (C) | 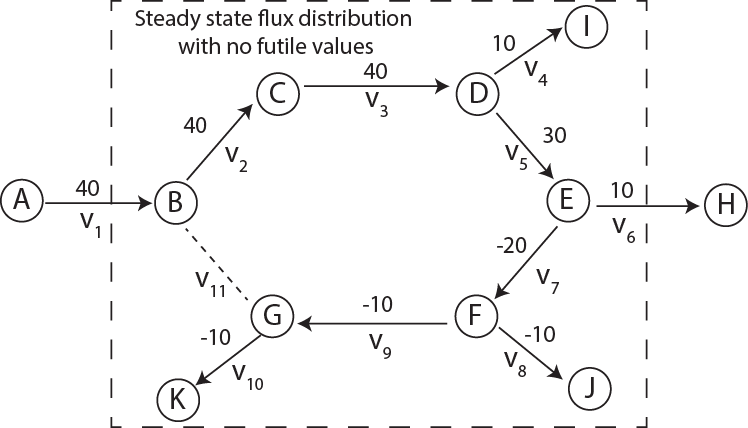 |

Figure S1: A toy network with loop for illustrating the method to remove futile flux values. (A) The structure of the network and the reaction bounds are represented graphically. Reactions v2, v3, v5, v7, v9 and v11 form a loop. Reaction v4, v8, v10 are the constraint. v1 is the exchange reaction providing source for the network. v6 is the reaction that should be maximized. (B) FBA returns a solution that contains a loop and an objective value of 100. To eliminate the less meaningful flux values in this case, v11 is constrained to zero as it has the lowest absolute flux value. (C) By eliminating reaction v11, a biological meaningful flux values for v2, v3, v5, v7 and v9 are obtained without degenerating the objective of 100.

In the network, the route from A to H is an optimal flux vector, but a cycle exist in the toy network in which reactions at different rates do not influence (i.e., enhance or reduce) the yield of H on A at a steady state (Figure S1 (B)).

This method may overlook some hidden futile cycles, since in some cases those futile cycle reactions could have flux rates within a reasonable range (e.g., the absolute flux value is less than 100 mmol/gDW/h) and appears to be ‘normal’.

In the case of the total NADH consuming flux as a function of biomass production rate under the MET mode, the reactions that were selected to be constrained to 0 were summarized in Table S1 according to the simple method aforementioned. Elimination of the fluxes through these reactions did not change the growth rate and the net NADH production rate in the MET mode, but help obtain a more feasible maximum total NADH consuming flux associated with growth and non-growth maintenance. However, since the hidden futile cycle might still exist in the network, the method did not guarantee that all of the cycle reactions were identified in the FBA result.

Table S1: Three sub-sets of reactions constrained to zero for eliminating the activated reactions of futile cycles.

|  | ID | Name | Reaction |
| --- | --- | --- | --- |
| A | GLUDx | glutamate dehydrogenase (NAD) | [c] : glu-L + h2o + nad <==> akg + h + nadh + nh4 |
|  | MDH | malate dehydrogenase | [c] : mal-L + nad <==> h + nadh + oaa |
|  |  |  |  |
| B | GLUDx | glutamate dehydrogenase (NAD) | [c] : glu-L + h2o + nad <==> akg + h + nadh + nh4 |
|  | MDH | malate dehydrogenase | [c] : mal-L + nad <==> h + nadh + oaa |
|  | G3PD1 | glycerol-3-phosphate dehydrogenase (NAD) | [c] : glyc3p + nad <==> dhap + h + nadh |
|  |  |  |  |
| C | GLUDx | glutamate dehydrogenase (NAD) | [c] : glu-L + h2o + nad <==> akg + h + nadh + nh4 |
|  | MDH | malate dehydrogenase | [c] : mal-L + nad <==> h + nadh + oaa |
|  | G3PD1 | glycerol-3-phosphate dehydrogenase (NAD) | [c] : glyc3p + nad <==> dhap + h + nadh |
|  | ME1x | malic enzyme (NAD) | [c] : mal-L + nad --> co2 + nadh + pyr |

Table S2: the sub-set of constraints chosen for different modeling.

| Group of the constraints | COI |
| --- | --- |
| C | 0 |
| B | 1 |
| B | 2 |
| C | 20 |
| B | 100 |
| C | 200 |
| A | 400 |
| C | 900 |
| A | 1500 |
| C | 3000 |
